# Exonisation of an *Alu* element in the 3’-UTR contributes to SRD5A2 deficiency

**DOI:** 10.1038/s41598-026-64581-x

**Published:** 2026-08-04

**Authors:** Ralf Werner, Anna Basina, Axel Künstner, Sudharsan Agas Chandra Prakash, Kübra Kösem, Alexandra Kulle, Nadine C. Hornig, Stefan A. Wudy, Michaela F. Hartmann, Paul-Martin Holterhus, Hauke Busch, Olaf Hiort

**Affiliations:** 1 Division of Paediatric Endocrinology and Diabetology, Universität zu Lübeck, 23562 Lübeck, Germany; 2 Group of Medical Systems Biology, Lübeck Institute of Experimental Dermatology, Universität zu Lübeck, Lübeck, Germany; 3 Department of Pediatric Oncology and Rheumatology, Paediatric Endocrinology and Diabetes, University Hospital of Schleswig-Holstein, UKSH, Campus Kiel, Kiel, Germany; 4 Institute of Clinical Chemistry, UKSH, Kiel/Lübeck, Germany; 5 Institute of Human Genetics, Christian-Albrechts-University Kiel and University Hospital Schleswig-Holstein, Kiel, Germany; 6 Steroid Research and Mass Spectrometry Unit, Laboratory for Translational Hormone Analytics, Pediatric Endocrinology and Diabetology, Center of Child and Adolescent Medicine, Justus Liebig University, Giessen, Germany; 7 Present Address: Hormone-Hamburg, Hamburg, Germany

**Keywords:** Alu exonisation, Nonsense-mediated decay, Differences of sex development, SRD5A2 deficiency, Diseases, Genetics, Molecular biology

## Abstract

Steroid 5α-reductase deficiency is a rare autosomal recessive condition caused by mutations in the *SRD5A2* gene that leads to a severe virilisation deficit of the external genitalia in individuals with a 46,XY karyotype. Here we report an adult 46,XY person with clinically confirmed steroid 5α-reductase deficiency. Sanger sequencing revealed a compound heterozygous, maternal, pathogenic c.692A > G; p.(His231Arg) variant and a very rare paternal c.*66T > G variant in the 3’-UTR. RT-PCR products of individual’s derived genital skin fibroblasts revealed that only the maternal variant is expressed while the paternal variant could not be detected. PacBio RNA Isosequencing revealed that the variant c.*66T > G introduces a new strong splice donor site 66 nt after the canonical stop codon that is spliced to an inverted *Alu* sequence located 15 kb downstream. In all paternal, but none of the maternal transcripts, we found a new exon junction 66 nt after the stop codon within the 3’-UTR. Mammalian transcripts with an intron excision site > 55 nt downstream from a termination codon are subject to degradation by the nonsense-mediated decay (NMD) pathway. We conclude that the pathogenic maternal variant, together with the NMD-triggered downregulation of the paternal allele, is causative for the observed SRD5A2 deficiency.

**Supplementary Information:**

The online version contains supplementary material available at 10.1038/s41598-026-64581-x.

## Introduction

Steroid 5-alpha-reductase 2 (SRD5A2) deficiency (OMIM #264600) is a rare autosomal recessive condition leading to an impaired conversion of testosterone into the more potent androgen dihydrotestosterone (DHT)^1^. SRD5A2 deficiency in 46,XY individuals leads to a broad spectrum of 46,XY differences in sex development (DSD) ranging from typical female external genitalia, clitoromegaly, and ambiguous genitalia to a micropenis or hypospadias at birth. This variability is also observed among affected individuals carrying the same *SRD5A2* variation^2^. At puberty, a significant virilisation without gynecomastia is observed^3^ and often leads to a male gender reassignment. Gender change rates vary significantly across countries and ethnicities, ranging from 16% to 70%, independent of the virilisation score^4^. Within the last two decades, molecular diagnoses have influenced gender assignment and led to an increased assignment of affected newborns as males and lower gender change rates^4,5^. Therefore, accurate molecular diagnosis is key for good clinical management of SRD5A2 deficiency.

*Alu* elements belong to the group of short interspersed elements (SINE) and are non-autonomous, primate-specific, transposable elements. These elements are approximately 280 bp long, followed by a polyA-tail of variable length^6^. The human genome contains more than one million copies of *Alu* elements and SINEs, making up to 13% of the genome^7^. Many studies have described splice-mediated incorporation of parts of *Alu* elements from introns into mature mRNA^8^. Most of them introduce a frameshift or a premature termination codon and occur at low frequencies. Splice site mutations can activate cryptic splice sites of *Alu* elements, leading to predominant or exclusive *Alu* exonisation and conditions like ornithine δ-aminotransferase deficiency, Alport syndrome, or Dubowitz-like Syndrome^9–11^. On the other hand, alternative splicing of *Alu* elements can lead to new protein isoforms and enhance evolutionary genetic diversity^12^. Intergenic *Alu* exonisation facilitates the evolution of tissue-specific transcript ends, but can also compete with alternative splicing and may alter mRNA stability^13^. The effects of polymorphic and rare *Alu* insertion variants on diseases in the population are still relatively unexplored^14^. Here we describe a rare variant in the 3’-UTR of *SRD5A2*, 66 nt after the canonical stop codon, that leads to the exonisation of an intergenic *Alu* element localized 15 kb downstream of the stop codon, as a new disease mechanism in DSD.

## Results

The child was born with a phenotype suggestive of DSD. A hernia surgery at the age of 6 weeks on the right side showed a testis in the groin and led to further molecular analysis. Chromosomal analysis revealed a structurally normal 46,XY karyotype. Initial sequencing of the androgen receptor (AR), as well as an androgen binding assay with genital skin fibroblasts, excluded androgen insensitivity. In contrast, determination of 5 α-reductase activity from cell homogenates of the patient-derived cultured genital skin fibroblasts revealed a strong loss of 5α-reductase activity. Patient-derived 5α-reductase activity values were obtained on two different days and revealed values of 2.8 pmol/mg protein/h and 0.3 pmol/mg protein/h (reference values > 50 pmol/mg protein/h), indicating a strong loss of 5α-reductase activity. Steroid metabolome analysis of a 24 h urinary specimen revealed the typical metabolic pattern observed in SRD5A2-deficiency with subnormal excretion of 5α-reduced C_19_- and C_21_-metabolites reflecting the diagnosis of SRD5A2-deficiency (Supplementary figure S1).

**Fig. 1:**
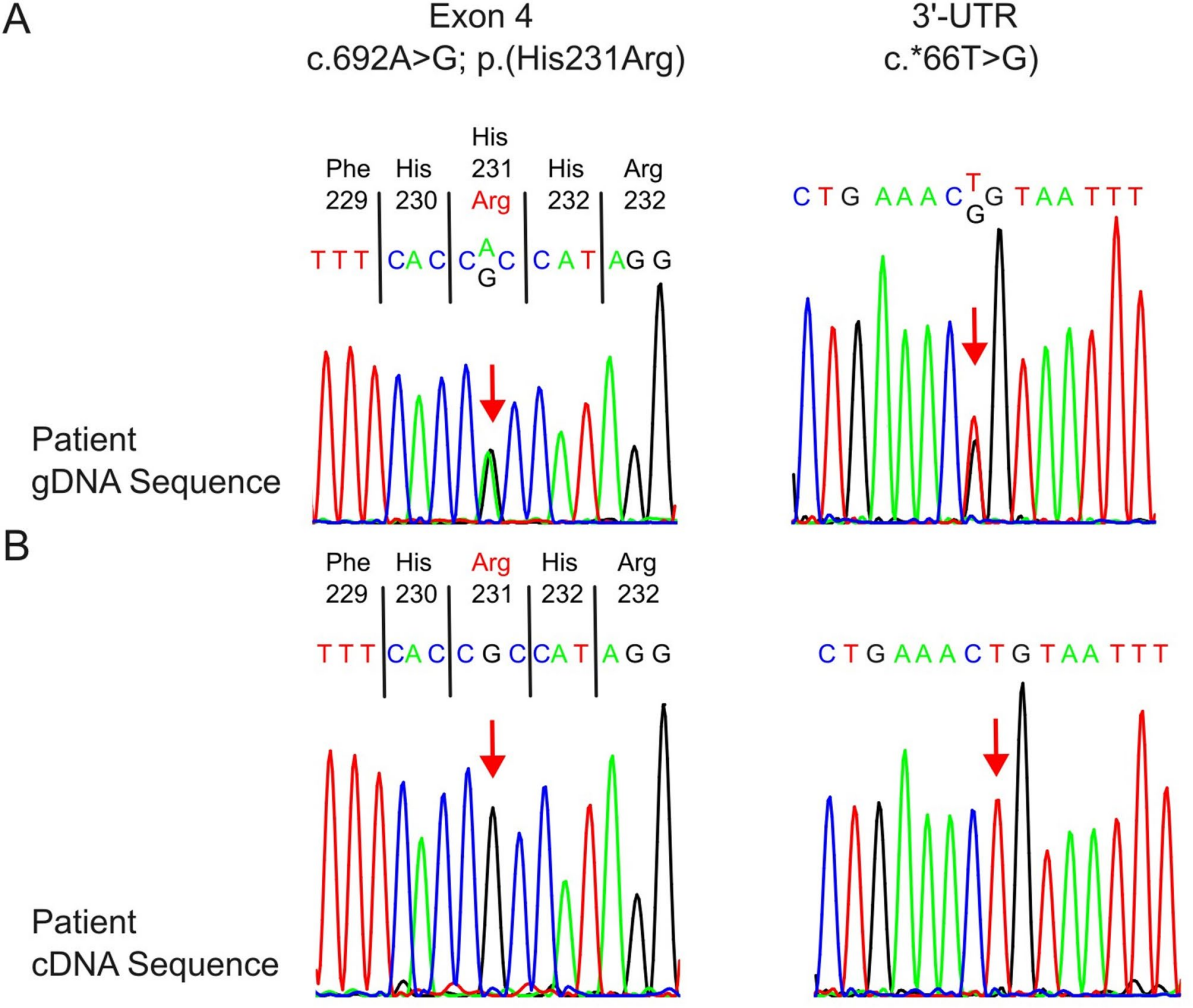
Sanger sequencing of the SRD5A2 variants. (**A**) DNA sequencing shows the compound heterozygous pathogenic variant c.692A > G in exon 4, inherited by the mother, and the rare 3’-UTR variant c.*66T > G, inherited by the father. (**B**) Sequencing of the cDNA from patient-derived GSF shows only expression of the maternal allele.

Subsequent Sanger sequencing of the *SRD5A2* gene revealed only a heterozygous mutation in exon 4 of *SRD5A2* (NM_000348.3:c.692A > G; NP_000339.2 p.(His231Arg)). This mutation was inherited from the mother and is a recurring deleterious variant associated with SRD5A2-deficiency and abnormal testosterone binding^15^. Since SRD5A2-deficiency is an autosomal recessive inherited trait, a second mutation in the paternal allele was suspected. The only rare mutation we could identify by Sanger sequencing was a T to G transversion located in the 3’-UTR, 66 nt after the stop codon (Fig. 1A, NM_000348.3:c.*66T > G; NC_000002.11:g.31751200 A > C). Analysis of the parental genomic DNAs (gDNA) revealed a paternal inheritance (Supplementary figure S2, A-C). This variant is extremely rare and was observed only once in a 46,XX female (allele frequency of 1.01 × 10^− 6^ (1 allele count/ 991,164, gnomAD v4.1.0, SNV:2-31526130-A-C (GRCh38) assessed April 15, 2025). To analyse the impact of this variant on the *SRD5A2* gene expression, we cultivated genital skin fibroblasts (GSF) derived from the patient and collected the total RNA for subsequent RT-PCR analysis. We performed exon spanning RT-PCR of exons 4 and 5, followed by direct sequencing of the amplicon, embedding the variants of both parents (maternal c.692 A; p.(His231Arg) and paternal c.*66T > G). Surprisingly, Sanger sequencing of the amplicon solely identified the maternal variant in exon 4 in a homozygous fashion, while any paternal variant was missing (Fig. 1B).

We expected a possible deep intronic splice variant that may have led to mis-splicing and would result in nonsense-mediated decay (NMD). Therefore, we performed whole-genome sequencing (WGS) of the patient’s DNA^16^(Supplementary methods). Analysis of the whole TAD-structure spanning approximately 590 Kb, enclosing the whole *SRD5A2* gene, its promoter and enhancer regions, as well as the 3’-UTR, did not detect any rare mutation that may affect transcription nor a newly generated putative splice site. The only rare *SRD5A2* mutations found were the maternal and paternal variants detected previously by Sanger sequencing (Table 1).

**Table 1:** Rare *SRD5A2* variants detected in the patient.

Inheritance	Exon	cDNA variant	Protein variant	Allele number gnomAD v4.1.0	Allele frequency	Germline classification
Maternal	4	c.692 A > G	p.(His231Arg)	460/ 1,613,614	2.85 × 10^− 4^	Pathogenic
Paternal	5	c.*66T > G	p.=	1/ 991,164	1.01 × 10^− 6^	Not known

Small RNAs loaded to an RNA-induced silencing complex (RISC) can bind to the 3`-UTR of target mRNAs and lead to destabilisation and degradation of the target RNAs^17^. This prompted us to analyse if the 3`-UTR mutation c.*66T > G in *SRD5A2* might create a new miRNA target site that could lead to a RISC-dependent degradation of the paternal mRNA and downregulation of *SRD5A2* expression. A custom search in the miRNA database (miRDB)^18^ did not show any match of known miRNAs to the putative new target site. Instead, we found putative miRNA binding sites that match the *SRD5A2* wt-sequence at this site (hsa-miR-6504-3p, hsa-miR-10399-5p, seed location c.*71 − 65). Both miRNAs share the same seed sequence. Therefore, we cloned the 3’-UTR variants in a pmirGlo luciferase vector to analyse the effect of the c.*66T > G variation on luciferase expression in a dual luciferase assay in HeLa cells. We cloned either a 56 nt long oligo that centres the c.*66T/66G variations or the 1610 nt long 3’-UTR of the paternal or maternal allele downstream of the firefly luciferase, but could not detect a significant effect of the paternal variant on luciferase expression (Supplementary methods, supplementary figure S3).

Next, we deep-sequenced the patient’s derived GSF poly(A) mRNA, yielding over 109 million reads, in order to more thoroughly analyse the expression of parental *SRD5A2* alleles. Surprisingly, we found a newly generated splice site at the paternal variation site spliced to an inverse *Alu* element located 15 kb downstream of the splice site, while all maternal alleles showed a normal 3’-UTR and polyadenylation at two major sites 1.6 kb and 3.7 kb after the stop codon. At the paternal splice site, we detected 28 reads, all spliced to an inverse *Alu*Jr element, suggesting a constitutive *Alu* exonisation in the paternal transcript. In contrast, at the same position, we detected 233 reads of the maternal allele. Comparing the distribution of maternal (233 reads, 89%) and paternal (28 reads, 11%) transcripts, it is possible that the maternal RNA coding for the harmful protein is more stable than the paternal RNA with the intact reading frame (Supplementary figure S4).

**Fig. 2:**
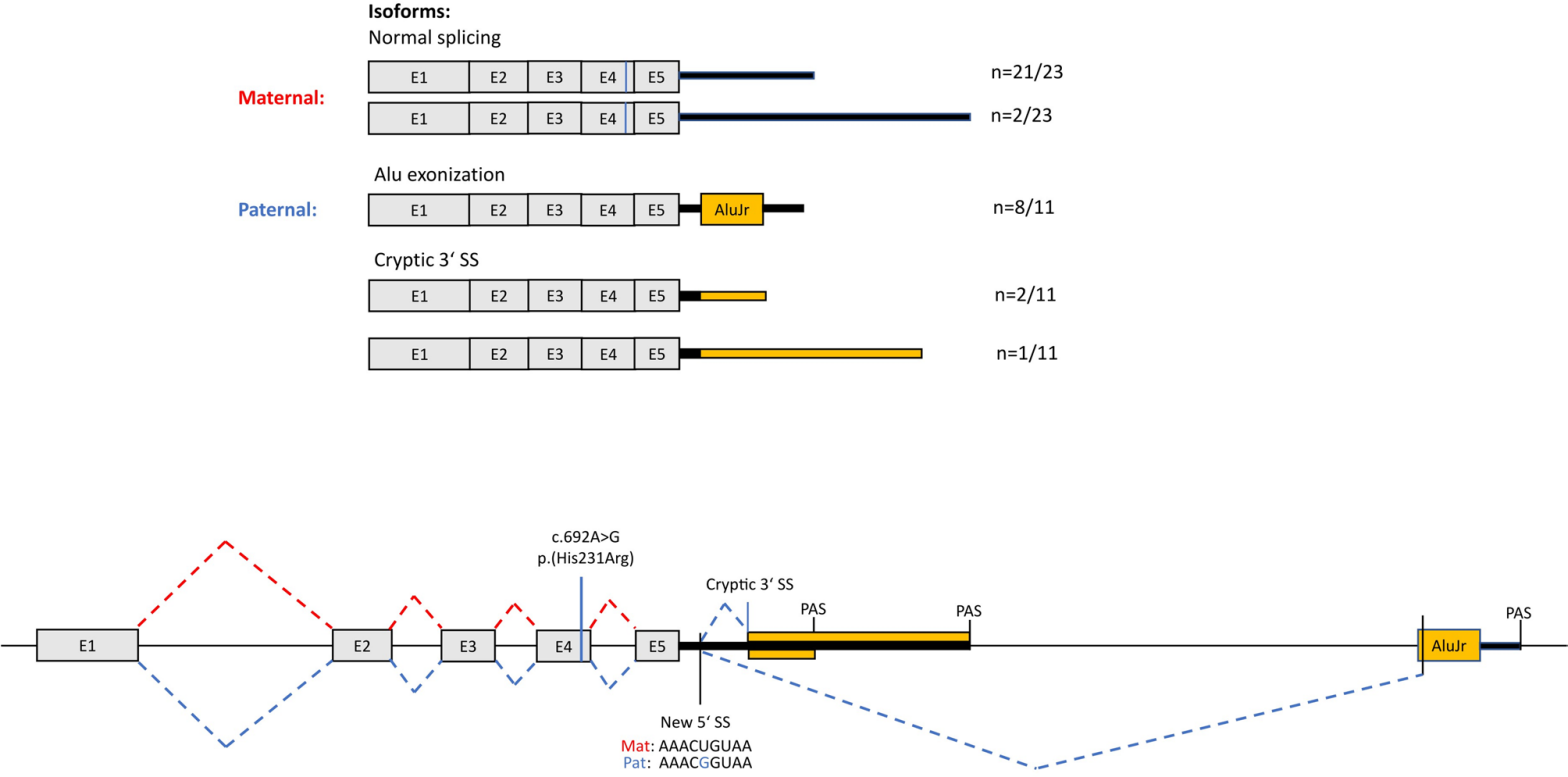
Full-length non-chimeric *SRD5A2* isoforms revealed by PacBio Isoform sequencing. PAS: polyadenylation site, SS: splice site.

To get a better overview of the *SRD5A2* isoform expression of both alleles, we performed isoform sequencing from total genital fibroblast RNA using the single-molecule real-time (SMRT) sequencing technology of Pacific Biosciences (PacBio). This technique allows sequencing full-length transcripts from the 5’-end to the poly(A) tail in real time and enables accurate detection of isoforms. SMRT sequencing detected 34 full-length non-chimeric reads (FLNCs, 23 mat; 11 pat). All paternal transcripts (*n* = 11) were spliced in the 3`-UTR, which is supposed to lead to the deposition of a new exon junction complex 66 nt downstream of the stop codon. Most paternal transcripts (8 out of 11) were spliced to the *Alu*Jr element 15 kb downstream. Three of them were spliced to another cryptic splice acceptor 860 nt downstream in the 3’-UTR of *SRD5A2*. Two of those show usages of the early polyadenylation site. The third transcript uses the late polyadenylation site. None of the maternal transcripts showed a splicing in the 3’-UTR. Both, the early and the late polyadenylation sites are used (Fig. 2).

## Discussion

The clinical diagnosis of the three most common causes of non-syndromic 46,XY DSD, SRD5A2- deficiency, androgen insensitivity syndrome, or NR5A1-haplo-insufficiency can be difficult due to a phenocopy of newborn to prepubertal patients and similar endocrine findings. Therefore, molecular genetic analysis should be considered as a first-line test^19–22^. Sanger sequencing of *SRD5A2* gene revealed a maternally inherited pathogenic heterozygous mutation within the reading frame and a very rare paternally inherited c.*66T > G variation in the 3`-UTR of unknown significance (VUS). Analysis of *SRD5A2* expression in patient`s-derived GSF by RT-PCR revealed only the presence of the maternal allele’s cDNA. Recently, a 3’-UTR mutation (c.*22C > A) in the *GFPT1* gene was described in patients with congenital myasthenic syndrome, creating a miRNA target site for miR-206*^23^. Therefore, we suspected that the very rare c.*66T > G variant might also create a new miRNA binding site that led to degradation of the paternal *SRD5A2* mRNA. We could not identify a miRNA that can bind specifically to the variation site of the paternal allele, but interestingly found two miRNAs (with the same seed sequence) matching the maternal allele’s site. Since there might be other undetected small RNAs that could lead to specific degradation of the paternal *SRD5A2* mRNA, we performed luciferase assays, but could not detect a significant downregulation of the paternal allele variant. We only observed a slightly lower (non-significant) expression of the maternal variant, which might be an effect of the matching miRNAs hsa-miR-6504-3p or hsa-miR-10399-5p, but their expression in HeLa cells has not been shown so far.

Subsequent deep sequencing of patient’s-derived GSF mRNA showed a splicing of the paternal 3’-UTR to an *Alu* element 15 kb downstream of the stop codon. Parts of *Alu* elements can be inserted by splicing into mature mRNAs, predominantly in their antisense orientation^8,24^. It has been shown that 5% of human alternatively spliced exons are *Alu*-derived and that internal exons containing *Alu* sequences are predominantly, if not exclusively, alternatively spliced^8^. Exonisation of a partial or complete *Alu* element that appears within the coding regions is likely to introduce a premature termination codon or frame shift^8^. In our case the introduction of a cryptic splice site by a rare variation 66 nt after the stop codon leads to exonisation of an antisense *Alu*Jr element and skipping of almost the full *SRD5A2* 3’-UTR including all polyadenylation signals (PAS) (Fig. 2). It has been shown previously that intergenic *Alu* exonisation can compete with alternative splicing and polyadenylation of the upstream gene and that intergenic *Alu* exonisation can contribute to tissue-specific posttranscriptional regulation^13^. The authors demonstrated that knockdown of the *Heterogenous Nuclear Ribonucleoprotein C* (*HNRCPC*) gene can lead to skipping of the terminal exon and *Alu* exonisation, or activation of a cryptic splice site in the 3’-UTR and *Alu* exonisation in the *Regulator of Microtubule Dynamics 3* (*RMDN3)* gene. Skipping of the terminal exon and *Alu* exonisation in the *RMDN3* gene could also be observed in the isoform sequences of our patient in one of 19 *RMND3* transcripts (Supplementary figure S5). The same is true for the rare exon 6 skipping and partial *Alu*Y exonization in the *NSUN2* gene (1 of 16 FNLCs, Supplementary figure S6). Mutational disruption of the canonical splice acceptor site in exon 6 of *NSUN2* leads a strong *Alu* exonisation and subsequent mRNA instability associated with a Dubowitz-like syndrome^11^.

In our case, the splicing mediated exonisation of the *Alu*Jr element, that is located 10 and 12 kb downstream of the genuine *SRD5A2* polyadenylation signals (PAS), leads to a skipping of these PAS. *Alu* elements contain PAS on its forward strand and not on its reverse strand^25^. Therefore, a PAS downstream the inverted *Alu*Jr sequence must be used for polyadenylation, since the bulk RNAseq as well as the isoform RNAseq were performed using oligo-dT primers for reverse transcription. Isoform RNAseq allowed us to accurately determine the strong PAS (AUUAAA) 1027 nt downstream the *Alu*Jr splice junction with polyadenylation 12–21 nt after the PAS (Supplementary figure S7).

**Fig. 3:**
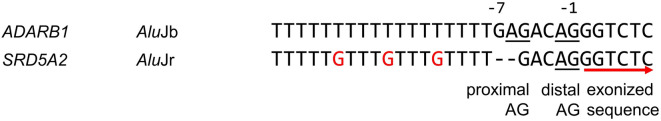
Comparison of the exonised *Alu*Jb splice site sequence in the ADARB1 gene (also named ADAR2) with the *Alu*Jr sequence exonised in the *SRD5A2* 3’-UTR. The *Alu*Jr sequence contained only the distal splice acceptor site and an interrupted polyU tract.

Two 3`-splice sites (3’SSs) of inverted *Alu* sequences are mostly used for *Alu* exonisation and are located at pos. 279 (proximal AG) and 275 (distal AG) of the *Alu* element. The distal AG is mainly used for exonisation of *Alu*J families^24^. Sequence comparison of the exonised *Alu*Jb sequence in the *ADARB1* gene (also named *ADAR2*) with the *Alu*Jr sequence exonised in the *SRD5A2* 3’-UTR revealed that only the distal AG is retained in the *Alu*Jr sequence and directly followed by a partially mutated tail polyadenylation site (TTTTTTGTTTGTTTGTTTT) while deletion of two nucleotides (-5G, -6 A) disrupted the proximal splice site (Fig. 3).

Since single mutations can activate the exonisation of a silent *Alu*, deletion of the proximal splice site may be sufficient to turn the distal AG to a strong splice acceptor^24^. Zarnack and colleagues have shown that hnRNP C safeguards the transcriptome from exonisation of *Alu* elements by binding to the U-tract at the 3`-splice site and preventing recognition of the *Alu* element through U2 Small Nuclear RNA Auxillary Factor 2 (U2AF65). Mutations disrupting the U-tract, similar to those observed here (Fig. 3) prevent hnRNP C recognition of the *Alu* element and can cause aberrant splicing of the *Alu* element^26^. Together with the c.*66T > G variation that creates a constitutive splice donor site, this may lead to a strong exonisation of the *Alu* sequence. Using long-read isoform sequencing, we also found a minor activation of a cryptic splice acceptor (3/11 reads) 860 nt downstream of the c.*66T > G site (Supplementary figure S8). Nevertheless, all paternal transcripts without any exception were spliced at the c.*66G splice donor site. Exonisation of an *Alu* element in the 3’-UTR, as well as removing an 860 nt intronic sequence from the 3’-UTR could lead to the deposition of an exon junction complex (EJC) just upstream of the splice junction. Transcripts harbouring an EJC > 55 nt downstream of a termination codon are subject to nonsense-mediated decay (NMD) that occurs as a result of interactions between the terminating ribosome, UPF1-3, and the EJC^27^. Although not experimentally proven here, NMD is the most likely cause of degradation of the paternal *SRD5A2* allele in our case and is corroborated by the quantification of paternal (28 reads, *Alu* exonised, 11%) vs maternal (233 reads, genuine polyadenylated, 89%) transcripts in bulk RNAseq (Supplementary figure S4).

Interestingly, SpliceAI^28^, a deep neural network that enables precise prediction of noncoding genetic variants causing cryptic splicing, revealed a splice donor gain for variant chr2-31526130-A-C with a Δ score of 0.78, but could not identify the acceptor site, most likely due to its remote location (15 kb downstream). Cryptic splice site activation has rarely been described as a cause of DSD, mostly affecting AR (androgen receptor) expression, causing androgen insensitivity syndrome^29–33^. Recently, a variant 152 nucleotides after the canonical stop codon in the kelch-like family member 40 (KLHL40) gene has been shown to induce Nemaline myopathy 8, likely by altered splicing in the 3’-UTR provoking nonsense-mediated decay^34^. Using SpliceAI, Jaganathan and coworkers estimated that 9%-11% of pathogenic mutations in patients with rare genetic disorders are caused by activation of cryptic splice sites. Therefore, similar pathogenic mechanisms involving 3`-UTR splice donor creation and distal *Alu* exonisation may be underrecognised in other Mendelian disorders and warrant consideration during variant interpretation. To our knowledge, this is the first description of *Alu* exonisation leading to DSD. Furthermore, this report highlights the importance of non-coding variants as a genetic cause of DSD.

## Subjects and methods

### Patient description

The child was born at term from healthy parents at an outside institution. Initial presentation at birth showed ambiguous genitalia with a phallus of approximately 20 mm in length. An introitus vaginae was not visible. The labioscrotal fold was asymmetrical with a descended gonad on the left side. Ultrasound revealed the right gonad in the inguinal canal. A uterus was not seen. A vaginal pouch was seen with a low confluence to the urethra. At the age of 6 weeks, the right gonad was biopsied during an orchiopexy, and histologically, testicular tissue was seen. The karyotype was 46,XY. Laboratory diagnostics at this time were obtained at an outside institution and showed basal values for LH of 7.5 IU/l (ref: <0.5 IU/l), FSH 2.4 IU/l (reference range: 0.2–6.6 IU/l), estradiol 190.9 pmol/l (reference range: <55 pmol/l), testosterone 3.74 nmol/l (reference range: 0.035–6.14 nmol/l) and dihydrotestosterone 0.358 nmol/l. Three days after hCG-stimulation with 5.000 IE/m² the testosterone was measured with 9.36 nmol/l, androstendione 2.1 nmol/l and dihydrotestosterone with 0.79 nmol/l, respectively. The values were interpreted as typical for the mini-puberty and also as informative of testicular tissue being functional. Therefore, at this time, the suspected diagnosis was partial androgen insensitivity. Blood DNA was obtained and sent to our laboratory, where screening of the androgen receptor gene as well as the *SRD5A2*-gene with single strand confirmation analysis at this time did not show any variation^35^.

At the age of 4 years, further diagnostic procedures were initiated. A genitoscopy and laparoscopy confirmed the absence of a uterus. Both gonads had a typical testicular structure, and a ductus deferens was seen on both sides. Histology showed age-appropriate prepubertal testicular tissue. At this time, a genital skin biopsy was taken for further studies. A 5α-reductase activity assessment was performed in a cell homogenate at an outside institution and was measured with 2.8 pmol per mg protein/h (reference range > 50 pmol per mg protein/h), suggestive of 5α-reductase deficiency^36^.

The patient was followed until the age of 10 years. At this time, the gender was female, and the patient was in a prepubertal stage. At the age of 14 years, she was followed at an outside hospital. Her phallus had enlarged significantly at puberty from 2.5 cm to 4.5 cm. Therefore, she started on therapy with GnRH-analogues to suppress endogenous puberty. As the adolescent wished to remain in a female role, a therapy with estrogens and gestagens had been initiated. At this time, a GC-MS urinary steroid metabolome analysis was carried out, showing the typical metabolic constellation of 5α-reductase deficiency with subnormal excretion of 5α-reduced metabolites. This prompted us to re-sequence the *SRD5A2* gene, as the parents had permitted further use of any biomaterials and health data.

### Urine steroid diagnostics

Urine steroid diagnostics were performed using gas chromatography / mass spectroscopy^37,38^. In brief, 5 ml of urine underwent solid phase extraction, enzymatic hydrolysis, and derivatisation (formation of methyloxime-trimethylsilyl-ethers). An aliquot of the extract was subjected to GC-MS analysis (Agilent Technologies 6890 N GC housing a 25 m Optima-1 MS fused silica column (film 0.1 μm, inner diameter 0.2 mm) coupled to an Agilent Technologies 5975 inert XL mass selective detector). 34 urinary steroid metabolites were simultaneously determined using selected ion monitoring mode.

### Cell culture

Patient-derived genital skin fibroblasts (GSF) were cultured as described earlier^39^. Briefly, for RNA and steroid analysis assays cells were grown for 48 h without or within the presence of 30 nM or 300 nM testosterone in Dulbecco’s minimal essential medium with the nutrient mix F-12 (Sigma, Taufkirchen, Germany) supplemented with 10% charcoal-stripped fetal calf serum (PAA Laboratories GmbH, Coelbe, Germany), under 5% CO_2_ at 37° C. RNA was isolated using the RNeasy Mini Kit (Qiagen, Hilden, Germany). Cells were scraped in the presence of lysis buffer containing β-mercaptoethanol, passed through a Qia-shredder column, and the RNA was isolated according to the instructions of the manufacturer.

### Determination of 5α-reductase activity

5α-reductase activity was determined in two independent assays from cell homogenates of cultured genital skin fibroblasts^36^.

### Sanger sequencing

Sanger sequencing of the *SRD5A2* gene was performed for all 5 exons, including their splice sites. Primer sequences are given in supplementary table S1.

### RT-PCR

Total GSF-RNA was reverse transcribed using random primers and SuperScript II Reverse Transcriptase (Invitrogen, Life Technologies) according to the instructions of the manufacturer. A 406 bp fragment spanning exon 4 and exon 5 containing both, the positions of the mutation site c.692 A > G and the variant site c.*66T > G, was amplified using the primer pair 3`-UTR_66 fwd/rev (Supplementary table S2).

### Whole transcriptome analysis

Messenger RNA from patient-derived GSF was enriched from total RNA using oligo(dT) magnetic beads. After fragmentation, first-strand synthesis was performed using random hexamer primers. To allow directional sequencing of the library the second-strand synthesis was conducted using dUTP instead of dTTP. After end repair and A-tailing adaptors were ligated and the second strand cDNA was removed using uracil DNA glycosylase and endonuclease VIII (USER enzyme). After size selection and PCR amplification, the library was sequenced as 150 bp paired reads on an Illumina platform.

### Iso-Seq RNA-analysis

Messenger RNA from patient-derived GSF (RIN: 9.8) was reverse transcribed using the Iso-Seq Express 2.0 kit (PacBio) and used as input for the Kinnex full-length RNA kit (PacBio) to increase the library throughput on the PacBio sequencer. We obtained 14.9 million full-length non-chimeric (FLNC) reads with a maximum read length of 9,361 nt and a mean read length of 1,626 nt. After clustering and elimination of redundant FLNC sequences 498,806 of 505,248 cluster consensus sequences (98.72%) were aligned to the human reference genome (GRCh38). Alignments of cDNA reads were visualized using the integrative genome viewer (IGV 2.8.9)^40^.

### Ethics approval

This study involves human participants and was approved by the Ethics Committee of the University of Lübeck ID: AZ 08–081, Investigation of the molecular pathogenesis and pathophysiology of disorders of sex development. Ethics Committee of the Christian- Albrechts- Universität zu Kiel ID: D 410/08, ID: AZ 17–219. The study was performed in accordance to the Declaration of Helsinki. All participants and /or their legal guardians gave written informed consent to participate in the study before taking part.

## Supplementary Information

Below is the link to the electronic supplementary material.

Supplementary Material 1

## Data Availability

The data supporting the results of this study are available in the article and Supplementary Information, or can be made available from the corresponding authors upon reasonable request, after approval of the institutional ethics committee. The pathogenic variants described in the paper are available at ClinVar under accession ID: SCV007540401. The bulk RNA-seq data for the SRD5A2 region is available at the European Nucleotide Archive (ENA) under the accession number PRJEB112279.
