# Supplementary material for: Exonisation of an Alu element in the 3’-UTR contributes to SRD5A2 deficiency
Source: Sci Rep. 2026 Aug 4;16:24030. doi: 10.1038/s41598-026-64581-x (PMC13438754; doi:10.1038/s41598-026-64581-x)
Supplement: Supplementary file 1 [file 41598_2026_64581_MOESM1_ESM.docx]

**Supplementary Information**

**Supplementary Methods**

**Whole Genome Sequencing**

Short-read whole genome sequencing (WGS) was performed as described in detail earlier (1). In brief, genomic DNA was randomly fragmented. After end repair, A-tailing and adapter ligation, the library was amplified by PCR and purified using AMPure beads. 150 bp paired-end sequencing was performed on an Illumina HiSeq platform and yielded more than 104 GB of raw data and an average sequencing depth of 35x.

For the bioinformatic analysis, we reanalysed whole-genome sequencing (WGS) data from a previously published study (1). The analysis focused on the topologically associating domain (TAD) encompassing approximately 590 kb, including the entire *SRD5A2* gene, its promoter and enhancer regions, as well as the 3′ untranslated region (UTR). The annotated variants (hg19, ANNOVAR annotations, see (1) for further details) were screened for rare variants, potential regulatory disruptions, and cryptic splice sites.

**Plasmids**

The pmirGlo Dual-Luciferase miRNA Target Expession Vector (Promega Corp., Madison, WI) was used to evaluate the effect of the paternal variant c.*66T>G by inserting the putative miRNA target site 3` of the firefly luciferase gene. Either a 56 bp long fragment that centres the putative miRNA binding site or the first 1610 bp of the *SRD5A2* 3`-UTR sequence (up to the first polyA signal) were inserted into the *Sac*I and *Xba*I sites of the multiple cloning site.

**Luciferase assays**

HeLa cells were maintained in Dulbecco’s minimal essential medium (DMEM) with the nutrient mix F-12 (Sigma, Taufkirchen, Germany) supplemented with 10% fetal calf serum (PAA Laboratories, Coelbe, Germany) at 37 °C and 5% CO_2_. For transfections, 50.000 HeLa cells per well were cultured in 24 well plates overnight. Cells were co-transfected using 250 ng pmirGlo vector with either wt or mutant oligo/3’-UTR using 0.75 µl FuGENE HD (Roche Applied Science, Mannheim, Germany) per well. Luciferase activity was determined 48 h post transfection using the Dual Luciferase assay (Promega Corp., Madison, WI) and normalized for transfection efficiency. Relative luciferase activity of the empty pmirGlo vector was set to 100%.

**Supplementary Tables**

Table S1: *SRD5A2* genomic Sanger sequencing primers

|  | Upper primer | Lower primer |
| --- | --- | --- |
| Exon 1 | 5`-GGCGCTCCATAAAGGGGTTGC | 5`-GGGGAGTGAAGGCGGCGTCTGT |
| Exon 2 | 5`-GTTAAGGCGAAATGGCAGAGC | 5`-TAGAGGTGAGGGAGGGGAAGATGG |
| Exon 3 | 5`-ACTCATTGCCCTCCCCACTTTCTG | 5‘-AGCCGATTCCAACCCTACTCAA |
| Exon 4 | 5`-AGGCCACTCTTCGCAACTCCAG | 5`-ACCTTCCCGGCCGTCACCTCA |
| Exon 5 | 5`-CGAAATAGTCAGGCCCAAAATAAC | 5`-TTGCCCGGTGAAAGACATAGGAGA |

Table S2: RT-PCR primers

| 3`-UTR_66_fwd | 5`-aggTggcTTgTTTacgTaTgTTTc |
| --- | --- |
| 3`-UTR_66_rev | 5`-TcccTggaagggTaggagTaaacT |

**Supplementary Figures:**

**Figure S1: Targeted GC-MS analysis of urinary androsterone and allo-tetrahydrocortisol as biomarkers of 5α-reductase deficiency.**
A) Selected ion monitoring (m/z 270) of urinary androsterone (An, 5α-reduced C_19_-steroid) and its 5β-isomer etiocholanolone (Et) in the patient with 5α-reductase deficiency (above) and a healthy control (below). The ratio An/Et (reference range 1.66 ± 0.61, mean ± SD,(2)) was significantly reduced in the patient (0.22) in comparison with the healthy control (1.63). B) Selected ion monitoring (m/z 652) of urinary allo-tetrahydrocortisol (aTHF, 5α-reduced C_21_-steroid) and its 5β-isomer tetrahydrocortisol (THF) in the patient with 5α-reductase deficiency (above) and a healthy control (below). The ratio aTHF/THF (reference range 1.25 ± 0.50, mean ± SD,(3)) was significantly reduced in the patient (0.05) in comparison with the healthy control (1.83).

**Figure S2: Parental inheritance of the *SRD5A2* variants.**

Sanger sequencing of patient-derived genomic DNA (A) revealed a heterozygous p.(His231Arg) variant in Exon 4, inherited by the mother (B) and a c.*66T>G variant, inherited by the father (C).

**Figure S3. The *SRD5A2* variant c.*66T>G did not reduce luciferase activity in a pmirGlo assay.** Neither a short 56 nt long oligo nor the full-length 3’-UTR containing the c.*66T>G variant cloned into a pmirGlo reporter vector reduced firefly luciferase expression 48h post transfection in HeLa cells. Expression values were determined in three independent assays at different days in triplicates. Error bars represent ± 1 SD. Relative luciferase activity of the empty pmirGlo vector was set to 100%.

**Figure S4. Deep sequencing of patient-derived GSF mRNA.**
A) Overview of splicing events in *SRD5A2*. B) All paternal transcripts containing the c.*66G variant are spliced to an *Alu*Jr sequence located 15 kb downstream (28 reads, 11%) while all maternal transcripts containing the c.*66T variant have a normal unspliced 3’-UTR (233 reads, 89%). Alignment of cDNA reads using the integrative genome viewer (IGV 2.8.9) and the hg38 reference genome (4).

**Figure S5. Skipping of the terminal exon and *Alu* exonisation in the *Regulator of Microtubule Dynamics 3* (*RMDN3*) gene.** Alignment of patient-derived cDNA reads using the integrative genome viewer (IGV 2.8.9) and the hg38 reference genome (4). One FLNC cDNA read of patient’s derived GSF showed skipping of the last exon and *Alu* exonisation.

**Figure S6: Rare skipping of exon 6 and partial *Alu* exonisation in the *NOP2/SUN RNA methyl-transferase family, member 2* (*NSUN2*) gene in one FLNC cDNA read of patient’s derived GSF.** Alignment of cDNA reads using the integrative genome viewer (IGV 2.8.9) and the hg38 reference genome (4).

**Figure S7: A strong polyadenylation signal (PAS, AUUAAA) is located 1027 nt downstream the *Alu*Jr splice junction with polyadenylation 11-20 nt after the PAS.** Alignment of patient’s GSF-derived cDNA reads using the integrative genome viewer (IGV 2.8.9) and the hg38 reference genome (4).

**Figure S8.** Skipping of exon 3 and 4 and splicing of paternal transcripts by activating a cryptic splice acceptor 860 nt downstream in the 3’-UTR of *SRD5A2*. Alignment of patient’s GSF-derived cDNA reads using the integrative genome viewer (IGV 2.8.9) and the hg38 reference genome (4).

**References**

1.	Meinel JA, Yumiceba V, Kunstner A, Schultz K, Kruse N, Kaiser FJ, Holterhus PM, Claviez A, Hiort O, Busch H, Spielmann M, Werner R. Disruption of the topologically associated domain at Xp21.2 is related to 46,XY gonadal dysgenesis. *J Med Genet*. 2022. (<https://doi.org/10.1136/jmg-2022-108635>)

2.	Remer T, Boye KR, Hartmann MF, Wudy SA. Urinary markers of adrenarche: reference values in healthy subjects, aged 3-18 years. *J Clin Endocrinol Metab*. 2005;90(4):2015-21. (<https://doi.org/10.1210/jc.2004-1571>)

3.	Wudy SA, Hartmann MF, Remer T. Sexual dimorphism in cortisol secretion starts after age 10 in healthy children: urinary cortisol metabolite excretion rates during growth. *Am J Physiol Endocrinol Metab*. 2007;293(4):E970-6. (<https://doi.org/10.1152/ajpendo.00495.2006>)

4.	Robinson JT, Thorvaldsdottir H, Winckler W, Guttman M, Lander ES, Getz G, Mesirov JP. Integrative genomics viewer. *Nat Biotechnol*. 2011;29(1):24-6. (<https://doi.org/10.1038/nbt.1754>)
